# A scalable data collection, characterization, and accounting framework for urban material stocks

**DOI:** 10.1111/jiec.13198

**Published:** 2021-09-25

**Authors:** Hadi Arbabi, Maud Lanau, Xinyi Li, Gregory Meyers, Menglin Dai, Martin Mayfield, Danielle Densley Tingley

**Affiliations:** 1 Department of Civil and Structural Engineering, The University of Sheffield, Sheffield, UK; 2 Department of Civil and Structural Engineering, Sir Frederick Mappin Building, Mappin Street, Sheffield, S1 3JD UK

**Keywords:** bottom-up analysis, building material stock, environmental modeling, industrial ecology, machine learning, mobile mapping

## Abstract

Building stocks represent an extensive reservoir of secondary resources. However, common bottom-up characterization of these, often based on archetypal classification of buildings and their corresponding material intensity, are still not suitable to adequately inform circular economic strategies. Indeed, these approaches typically result in a loss of building-specific details, and a building stock characterization in terms of material mass, for example, glass, rather than component, for example, window. To deliver this higher resolution of details, a scalable approach to urban stock characterization, that enables a bottom-up estimation of building stocks at the building component level, is needed. In this paper, we present a framework to automate the characterization of urban stock. By using and combining a mobile-sensing approach with computer vision, urban stocks can be captured as 3D surface maps allowing the identification and semantic classification of stock objects, components, and materials. We demonstrate the potential of this framework through a case study of a neighborhood in Sheffield, UK, by using a prototype workflow comprising a custom-made mobile-sensing platform and an existing suite of neural network models to calculate an estimate count of buildings external doors and windows. The prototype implementation of the framework achieves comparable total and building-level component counts with those achieved through manual human counts. Such automated estimation of components enables an understanding of opportunities across the circular economic hierarchies and informs stakeholders across the supply chain to better prepare for the implementation of circular strategies including building refurbishments.

**Supplementary Information:**

The online version of this article (doi:10.1111/jiec.13198) contains supplementary material, which is available to authorized users.

## INTRODUCTION

With 189 nations participating, the Paris Agreement encourages a reporting mechanism for emissions in an attempt to avoid global temperature rises of over 2°C (2015). Given that somewhere between 20% and 40% of emissions are attributable to the built environment, global material stock characterization and accounting is essential for decarbonization and avoiding an extreme climate change fallout (World Economic Forum & Boston Consulting Group, 2019). Achieving these climate goals will require radical action across nations, cities, and sectors, but progress has stalled. In the United Kingdom, for example, meeting the country’s new net-zero emissions ambition by 2050 will be a considerable challenge, that requires urgent action (Committee on Climate Change, 2019).

Decarbonization of the built environment must tackle two critical areas: (i) embodied impacts and (ii) operational impacts. The first is in part delivered by a shift to the circular economy, as this would reduce long-term material consumption. In addition to the critical roles the built environment plays in our socio-economic metabolism (Haberl et al., 2017), its stocks are also an extensive repository of secondary resources. Anthropogenic stocks have undergone a 23-fold increase and are still on the rise (Krausmann et al., 2017). These anthropogenic resource reservoirs are thus an opportunity for resource recovery through circular economic strategies, especially in urban areas, which are characterized by a dense accumulation of built environment stocks.

Material stock and flow accounting can assist in predicting future material demand based on the current stock age and likely replacement rates (Tazi et al., 2021). This is especially important for those nations with older building stocks, such as the United Kingdom, where 85% of the building stock which will exist in 2050 is predicted to have already been built (Edwards & Townsend, 2011). This also means that operational impacts need to be reduced through energy retrofit of the existing stock. Thus, the more we understand about this stock, the better it can be maintained and *mined* in the future.

### Building stock accounting

Characterizing built environment stocks can be done through two main approaches, namely top-down and bottom-up. The top-down approach relies on mass balance and lifetime distribution to model the accumulation of material stock within a system over time. This approach has proved useful to achieve an overview of stock dynamics over long periods of time, allowing the identification of patterns and drivers that can be used to benchmark future stock accumulation (Lanau et al., 2019). The bottom-up approach, although time intensive, is preferred when generating detailed information on the physical arrangement of the system under study (Recalde et al., 2008). Bottom-up approaches consist of counting a given stock *piece by piece*, differentiated in terms of materials and stock composition. Spatial differentiation of results can also be achieved through integration with geographical information systems (GIS). Such GIS-based bottom-up approaches were first used by Tanikawa and Hashimoto (2009), where they studied the accumulation of materials in the built environment of two neighborhoods in Manchester, UK and Wakayama, Japan. Since then, a number of studies have been conducted at different spatial scales, on all parts of the built environment (Augiseau, 2017; Lanau & Liu, 2020; Tanikawa et al., 2015) or specific parts, such as roads (Guo et al., 2014), subway (Lederer et al., 2016), pipe networks (Wallsten et al., 2013), and cable networks (Krook et al., 2011). A number of case studies also focus on solely characterizing building stocks of cities, such as Vienna, Austria (Kleemann et al., 2017); Esch sur Alzette, Luxembourg (Mastrucci et al., 2017); Grenada (Symmes et al., 2020); Padua, Italy (Miatto et al., 2017); Melbourne, Australia (Stephan & Athanassiadis, 2017); Chiclayo, Peru (Mesta et al., 2019), as well as nations such as Germany (Ortlepp et al., 2016, 2018).

### Delivering the circular economy

For the circular economy, the building stock has proved to be a critical part of the built environment as it hosts a wide variety of easy-to-access and easy-to-recover materials above the ground (Lanau & Liu, 2020). The implementation of a circular economy faces a number of challenges industry wide, including a limited awareness across the supply chain (Adams et al., 2017), concerns about the consistency of flows of returned goods, and an unclear market demand for secondary resources (Guldmann & Huulgaard, 2020). Overcoming such barriers requires highly detailed modeling of building stocks so that stakeholders throughout the supply chain can obtain the exact quantity and quality of secondary resources, for example, construction bricks or panes of glass, that would be recoverable from a specific building nearing demolition (Arbabi et al., 2020). To enable this, estimates of material quantity and quality need to be spatially explicit and be measured at a building level. However, because building inventory datasets are heterogeneous in terms of construction type, periods, and use, buildings are often classified into archetypes, according to their characteristics to homogenize the datasets resulting in a loss of detail. These geo-located and building-specific details are, however, crucial to circular economic strategies.

Another shortcoming of common bottom-up modeling of building stocks for circular economy is the quantification of stocks in terms of materials mass rather than building components. In a circular economic paradigm, component reuse is always preferable to material recycling (Hyman et al., 2013). Component-level information is critical when aiming to estimate circular economic potential of a building stock. The number of windows shows the potential for future replacement, remanufacturing, and reuse, whereas mass of glass only shows the scope for recycling (Arora et al., 2019). Achieving a truly high-resolution stock characterization would require inspection of individual stock buildings. Undertaking such inspections across whole cities can be prohibitively time intensive using current approaches.

The more recent uses of remote sensing can and has, to some extent, mitigated some of these associated problems (Mao et al., 2020). These, however, are still lacking in the resolution they offer in stock characterization, especially with respect to components at a building level (Peled & Fishman, 2021). Current archetype-based approaches to stock accounting require increasingly more detailed archetypes to increase precision of the results. This inherently poses a data collection challenge and raises the level of uncertainty at building level (Ortlepp et al., 2018). Barring a few choice countries, including Denmark and Germany, such inventories, or data required to simply assemble them, do not readily exist in many others (Lanau et al., 2019). Given the size of the challenge, that is, building-specific characterization of the entirety of the stock involving buildings numbering in the millions for a given city or nation, there is a need for a scalable approach to urban stock characterization that enables a bottom-up accounting of stock at component level.

## BUILDING STOCK CHARACTERIZATION: RELEVANCE, CHALLENGES, AND OPPORTUNITIES FOR A SCALABLE FRAMEWORK

We propose aframework integrating mobile-sensing approaches and workflow automation in urban stock modeling to start addressing the scalability in stocks characterization. First, we define scalability by borrowing a definition common to systems engineering (Bondi, 2000; Jogalekar & Woodside, 2000; Weinstock & Goodenough, 2006). Scalability of an approach is measured as the extent to which it can be repeatedly extended to handle increasing workload with an optimized cost-effectiveness and without additional resource penalties. The methods by which we characterize stocks would need to become both faster at a building level and more efficient at a city/country level. The use of mobile/remote-sensing, computer vision, and deep learning methods are likely candidates to achieve *scalability*.

### A scalable framework

Computer vision can be defined as the “task of learning the qualitative representation of visual elements in their raw form in order to quantify them” (Ibrahim et al., 2020). In the last decade, boosted by the development of deep learning, computer vision has become an efficient way of modeling different aspects of cities. With regard to the built environment, the applications of computer vision can be categorized into two groups: seeing cities from above and from street level (Ibrahim et al., 2020). Satellite remote-sensing approaches have focused on night-time light for the estimation of in-use stock of metals and other materials in infrastructures and buildings (Rauch, 2009; Takahashi et al., 2009, 2010; Liang et al., 2017; Peled & Fishman, 2021). Seeing closer to the street level, however, has not yet been used to inform building stock research extensively, despite the quickly maturing literature developed as part of the efforts undertaken developing autonomous vehicles (Zhang et al., 2020). So far, its applications include the assessment of land-use (Srivastava et al., 2019), urban vegetation cover (Seiferling et al., 2017), or the detection of potholes on roads (Dhiman & Klette, 2019).

**FIGURE 1:**
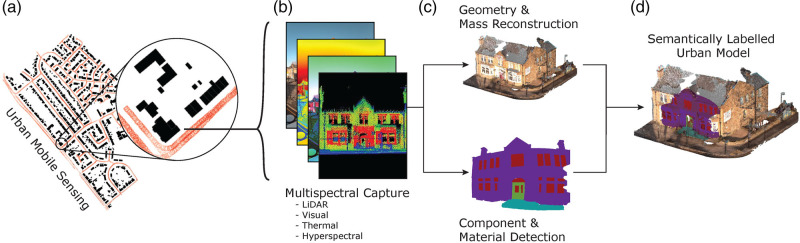
Framework schematic for a scalable spatially explicit and high-resolution urban stock characterization using multi-sensor mobile mapping (a, b) to enable reconstruction of building geometry and detection of components and materials (c, d) for a semantically labelled urban surface model (d)

As the overall framework in Figure 1 illustrates, a mobile-sensing approach with computer vision and machine learning to construct 3D urban surface maps would allow us to identify and classify stock objects, components, and materials specific to individual buildings. The framework consists of an initial data collection stage using a confederation of sensors. A suite of machine learning and computer vision approaches are then used to both reconstruct the 3D geometry of the urban scene and detect stock components and materials. Finally, the 3D reconstruction and detected components/materials are fused to generate a semantically labeled urban model that enables quantification of the stock components and material at a building level.

Our intentions in this work are not to develop/advocate a set of specific computer vision models for the sole purpose of stock accounting. The crux of the proposed framework rests in the argument for the integration of the mobile-sensing and computer vision approaches and use of automation to increase the pace and spatial resolution of stock accounting at a building/component level. The data streams suggested in the figure are, thus, informed by the existing literature on what currently can and ultimately could be extracted from each sensory capture: LiDAR enables accurate measurements of size and geometry of buildings and their facade components (Ackere et al., 2019), visual imaging satisfies minimum requirements to detect and recognize the facade components (Dai et al., 2021), and thermal and hyperspectral imaging enable detection of components’ material composition and wear condition (Ziolkowski et al., 2018; Yao et al., 2020; Cho et al., 2018).

### Data collection and management

The implementation of such a framework could experience some practical obstacles and/or policy implications. The main and immediate obstacle is ensuring a line-of-sight with buildings when using mobile-sensing approaches for data collection. For a complete implementation, the data captured would need to have full building coverage. Using only drive-by methods, for example, Google Street View or the case study in the next section, will result in quantifying only what the vehicle can observe from the street, leaving out the façade components that would generally be expected to be on the backside of the buildings or those on the front that are obstructed by vegetation or miscellaneous urban furniture. While this does not impact the validity of the information extracted for each building, a mixture of drive-by and fly-by imaging would be required to ensure full building envelope coverage to avoid systemic underestimation of components and stock. Drone-based fly-by building detection approaches are now fairly advanced in reconstructing building geometry from visual images (H. Huang et al., 2020). Remaining challenges in complementing drive-by methods with fly-by imaging are those relating to swarm dynamics. These would be crucial for scalability in the context of an automated deployment of a number of low-cost unmanned aerial vehicles to achieve the same pace and ease of en masse data collection as drive-by methods (Bouffanais, 2016).

A secondary group of implications would involve the collection, management, and governance of such integrated and automated mobile/remote-sensing frameworks. In terms of data collection, the proposed framework remains scalable, deployable, and manageable in terms of human effort and time needed to undertake the mobile sensing and maintaining the stock model. This inherently suggests potential for a public body or the academic community collectively shouldering computing costs and the management of such urban resources under an opensource open data framework. We should, however, note the high capital costs of developing and deploying a custom imaging vehicle with all four sensory requirements which is currently required for the implementation of the framework through drive-by imaging might limit development opportunities for commercial ventures. Although we currently do not take a position on a preferred overall collection or governance in reference to the implementation of the framework, we do believe the academic community engaged in developing and undertaking urban building stock accounting should engage in a dialogue on developing harmonized approaches to collection and reporting as already advocated by Heeren and Fishman (2019) in the case of material intensity surveys.

### Generalizability and scalability

An issue to further expand on is the suitability and limits of computer vision and machine learning for achieving a scalable approach to material stock characterization. The potential of these methods for increasing the speed at which stock accounting can be performed is more easily demonstrable given the progress made so far in image recognition in other engineering and medical applications (Brynjolfsson & Mitchell, 2017). The larger unknown is the generalizability potential of existing methods in the context of material accounting.

Building façade segmentation dates back a few decades. The current cutting edge of both building façade and the broader urban scene segmentation studies relies mostly on deploying and expanding methods using convolutional neural networks of various architectures (Badrinarayanan et al., 2016; Femiani et al., 2018; Fu et al., 2019; Zhao et al., 2017; Zolanvari et al., 2018; Schmitz et al., 2019). The community has also developed a series of public and often-used test datasets including ECP (Teboul et al., 2010) and Graz (Riemenschneider et al., 2012) for façade segmentation and Cityscapes (Cordts et al., 2016), Mapillary Vistas (Neuhold et al., 2017), and ApolloScape (X. Huang et al., 2018) for urban scene segmentation. The existing datasets for building façade and urban scene detection each pose their own particular challenges when adapted for purposes of building stocks accounting. In the context of scalability, these challenges concern the continued efficiency of models when applied to building components and geographic context outside the scope of the dataset. The building facade datasets often contain pre-edited images which affects the convenience of their use in training models that would be used with other mobile sensed images that will have a variety of viewing angles and conditions. The urban scene datasets, as mainly developed by the autonomous vehicle community, do not have this problem, but currently suffer from a lack of detailed labeling for components below a building level given their primary use case.

The immediate issues that need addressing for practical realization of the framework are those regarding the minimum size of the training sets needed to identify, with a reasonable accuracy, various building components within different national/geographic context and whether this differs between building components and across regions. Zhu et al. (2020) have recently worked on large-scale architectural asset detection in panoramic images across 17 different cities. Their work suggests that there are some essential qualities defining distinct building components. Although geographical proximity and architectural style, for example, windows frame structure, do provide for internal clustering of elements into subgroups with less internal variability. As such, as a starting point, existing façade detection models could be adapted in conjunction with visual imaging available from services similar to Google Street View. (See the online Supporting Information for a small demonstration on generalizability of façade components across geographic regions.) For practical uses, however, these models are likely to require some initial fine-tuning and retraining, not necessarily to compensate for differences in the building stocks used in original training, but for the embedded differences in the images due to variations in equipment, capture angles, etc.

### Integration with existing approaches

Given the methodological challenges set out in the previous sections, a scalable framework is still some ways off. On the path toward full implementation of such a framework, intermediary steps can be taken to make use of and maintain compatibility with the existing archetype-based methodological approaches. A high level of differentiation can be reached by using the archetypes as a base only and complementing it with as much information as possibly retrievable from computer vision. For example, most bottom-up approaches base their stock estimation entirely on archetypes. In fact, a hypothetical compromise between existing survey methods and the suggested framework could alternatively focus on training similar computer vision models to assign existing archetypes to buildings first. This could simply involve only the visual imaging of the building stock as already available through services such as the Google Street View. However, with the presented framework and utilizing models developed at component level, additional data will become available to refine results at building level, for example, areas of wall, windows, and doors, instead of being intrinsically estimated through an archetype-based approach as per Table Table 1.

**TABLE 1:**
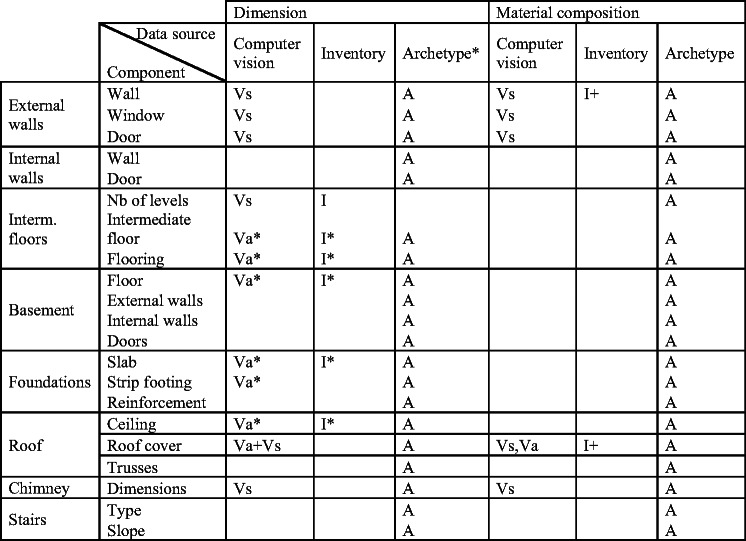
Typical information required to calculate building stocks with a bottom-up approach

For each component, information is needed on its dimensions and material composition. For each, several data sources can be used, depending on availability.

Abbreviations: I+, information only available if the inventory is detailed enough; Va, computer vision from above; Vs, computer vision from the street.

*The data source can give an indication, but further assumption might be required. This is the case, for example, with the area of a foundation slab: satellite data gives an indication of a building’s footprint that may be used as a proxy for the slab area if no other information is available.

Our framework can hence raise the precision of results while still keeping the data collection requirements feasible and scalable. Indeed, and most importantly, the main constraint in such a framework is the time required for the drive-by and/or fly-over sensing. This means that compared with existing bottom-up methods, the approach is more easily scalable across large urban areas. It is worth reiterating here that in this work, we are not advocating the use of a particular set of computer vision models or overall methodologies.

### Stock accounting for circular economy and global decarbonization

Arriving back at the broader potential of a scalable framework, the spatially explicit and component level understanding of material stocks is essential to deliver the circular economy. The circular economy is built on the idea of a continuous loop of materials, across multiple lifecycles, to reduce and eventually eliminate new resource extraction. Critical to the idea is that of maintaining materials at their highest value possible. In the case of the building stock, this would be maintaining materials together as a building in the first instance, meaning that building retrofit is a key part of the agenda. If buildings cannot be made fit for purpose, they should be deconstructed, and the components remanufactured or directly reused. Alternatively, they could be striped back into their component parts and the individual materials reused or more likely in this case recycled. The eventual full implementation of the framework enables derivation of façade materials quantity and quality. For example, the number of bricks that could be salvaged for reuse, or the mass of concrete available for downcycling. These building-specific observations can then be improved by linking in building archetypes to provide further information on what cannot be seen from the outside.

Addressing the larger decarbonization challenge involving buildings, a significant proportion of energy use in buildings is to maintain thermal comfort. Heat loss in older buildings is predominantly a function of the thermal performance of the building facade. This is driven by the material characteristics and method of construction. Our suggested framework would also facilitate a scalable identification of the stock most in need of retrofit. This is helpful to both predict future material demand and its carbon impacts, but also enables consideration of whole life carbon payback times for different insulation types (Li & Tingley, 2021; Moncaster et al., 2013) and could facilitate local authorities in bulk procuring retrofit interventions.

## PROOF OF CONCEPT: A PRELIMINARY CASE STUDY IN SHEFFIELD

In this section, we demonstrate the potential of the framework combining mobile sensing and computer vision in a small case study of a local neighborhood. This serves as a demonstration of how such a framework could be operationalized based on methodological components already available within the existing literature. The specifics of the neural network model used in the case study is of secondary importance in this paper.

The case study area is of roughly 2500 inhabitants spanning 2.79 km^2^ in the southwest of Sheffield, UK. In prototyping a demonstrator of the framework, we use a bespoke mobile-sensing platform and an existing cluster of neural network models to calculate an estimate count of the buildings’ external doors and windows. The mobile-sensing platform used, the multi-spectral advanced research vehicle, is a van mounted with a custom imaging rig, which enables collection of the four data streams required in the production of multi-spectral texturized 3D surface maps of the captured built environment (Meyers et al., 2019). For the case study presented here, we only make use of the visual imaging stream for demonstration purposes. (Interested researchers and groups should contact the corresponding author for data access.)

### Component and material detection for a semantically labeled urban model

**FIGURE 2:**
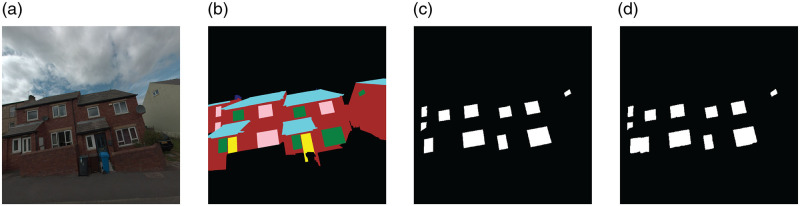
Sample of visual input to and output of the component segmentation model. (a) Raw image input, (b) sample annotation of façade component used for training, (c) sample window component truth mask isolated from the full annotation, and (d) sample model predicted window mask

In prototyping the component detection part of the framework, Figure 1c, we make use of an existing ensemble of convolutional neural networks trained to identify building components such as windows and doors at a pixel level (Dai et al., 2019), an example of which is shown in Figure 2. The incorporated ensemble of models is based on a U-Net architecture which segments input images, Figure 2a, for each component class separately, Figure 2b, before assembling the results together. The U-Net architecture was originally developed for processing and segmentation of medical images and uses a combination of high- and low-level image information to determine pixel-wise classification (Ronneberger et al., 2015), see Figure 2d.

We make use of the model developed by Dai et al. (2019) for this case study for convenience as it has originally been trained on output from the same research vehicle. The images used by Dai et al. are also captured in Sheffield, covering mostly terraced, semi-detached, and detached residential buildings, albeit in a different part of the city without any overlap with the stock used in the case study here. Dai et al. use an eight-category schema for labeling their training set comprising: background (non-building pixels), walls, roofs, chimneys, two variations of windows, and two variations of doors, Figure 2b. 240 images are then used for the training, validation, and testing of the model with a [80%, 5%, 15%] split of images across the training, validation, and test set. Data augmentation including horizontal flip, vertical and horizontal shifts, and hue adjustment is also applied to the images used by the authors (Krizhevsky et al., 2017).

All models are implemented and trained using existing TensorFlow libraries (Abadi et al., 2016). The results are then pooled together assigning the most confident pixel-level classification from among the categories to each pixel. Here, we should note that we do not retrain the model by Dai et al. specifically based on the data from the neighborhood used in this case study and treat their model as a black-box ready-made package. Further information regarding the specifics of the implementation and training of the ensemble of neural network models used including their accuracy and precision can be found in Dai et al. (2019).

For the purposes of our case study demonstration, we only use the models to detect and present counts for doors and windows since these are more inherently *countable* relative to, say, walls and roofs and as such more instructive in a wider discussion of the framework’s practical applications vis-à-vis circular economy and building refurbishments. Regarding broader questions on generalizability of the model used as raised in the previous sections, a toy example of the application of Dai et al. model to Google Street View images from a few different cities outside the United Kingdom is available in the Supporting Information.

### Other data and methods

The Ordnance Survey maintains and provides building footprint geometry for the United Kingdom (Ordnance Survey, 2020). These are obtained through a combination of aerial LiDAR and photographic imaging. The dataset, as such, includes building height as well as footprint geometry. Given that the focus of this paper is showcasing the potentials of a mobile and remote-sensing framework, we use this dataset here, as Ordnance Survey already provides the product. The same information, however, could have been obtained independently using the LiDAR capture from the mobile-sensing platform used for the visual imaging following existing methodologies (Vayghan et al., 2020; Wang et al., 2019).

**FIGURE 3:**
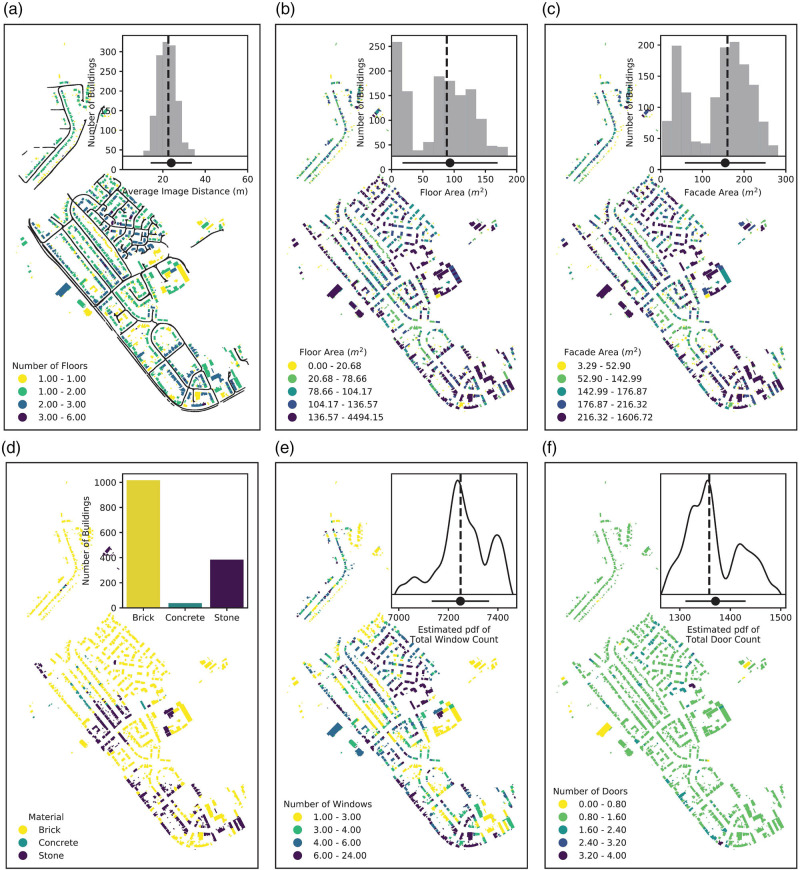
Spatial distribution of building characteristics and components. (a) Colormap of floor count along with the positions of the vehicle corresponding to the 42451 images. (b) Total floor area. (c) Façade area as estimated by height and footprint perimeter. (d) Predominant façade material. (e, f) Estimated numbers of windows and doors with insets showing the estimated probability density function of the expected value for the total neighborhood-wide count. For insets, dashed vertical lines denote the median with the point plots indicating mean and standard deviation. Note that colormap breaks are of different scales. Maps contain OS Map data © Crown copyright and database rights 2020. Data from this figure are available in File S2 of the Supporting Information

In order to obtain estimates of the count of façade windows and doors for each building, we first extract the 100 nearest vehicle positions, to the building centroid, and hence images taken looking in the building’s direction. The retrieved image positions and the distribution of average image distance from buildings can be seen in Figure 3a and its inset. Overall, we consider 42,451 unique photos for 1515 structural footprints within the neighborhood. (Raw images used are available from the corresponding author upon reasonable request.) The retrieved photos are then passed to the image segmentation models to extract the number of door and window components for each image. This results in distributions, each effectively based on a hundred sampled values of the count of doors and windows for each building. Since the field-of-view in each image is not limited to a single building, we scale the component count in each image by the number of buildings for which a given image has been retrieved and weigh them inversely with distance from the building such that

1 $$ \overline{{X}_{n}}=\left\lceil \frac{1}{100}\sum _{i}^{100}\frac{{x}_{i}}{{a}_{i}{l}_{i,n}}\, \right\rceil , $$

where $\overline {{X_n}}$ is the number of components, doors, or windows, for building $n$ with ${x_i}$ the number of components estimated for image $i$. ${a_i}$ is the number of buildings to which image $i$ is amongst the 100 nearest and ${l_{i,n}}$ is the distance between image $i$ and the centroid of building $n$. Note that the ceiling function avoids fractional component counts.

As the count of components are independent of one another, we estimate the mean and standard deviation of the total number of components across the neighborhood by estimating the sequential convolution of the buildings’ distributions of the component count following

2 $$ P\left( {\ {T_n} = t} \right) = \mathop \int \nolimits_{ - \infty }^\infty P\left( {{T_{n - 1}} = x} \right)P\left( {{X_n} = t - x} \right)dx, $$

where, $P( {{T_n}} )$ is the distribution function of the sum of component count, either door or window, of $n$ buildings, $P( {{T_{n - 1}}} )$ is the distribution function of the sum of component count of $n - 1$ buildings, and $P( {{X_n}} )$ is that of the component count for the $n{\rm{th}}$ building. We use Gaussian kernel estimates of the distributions of the doors and windows for each building in estimating the probability distribution of total number of components in the neighborhood.

### Building components and materials

Figure 3 shows the spatial distribution of the stock information extracted from the footprint, height, and mobile-sensing platform. The neighborhood is mostly made up of single or double story terraced and semi-detached houses with a handful of medium-rise apartment blocks, Figure 3a. Figures 3b and 3c show the distributions of building floor and façade areas. The bi-modal appearance of these is due to the inclusion of garages and temporary structures which comprise the peaks at the lower values. The rest of the buildings show an average floor and façade area of 100 and 150 m^2^, respectively. Assuming an average wall thickness of 300 mm, the mean neighborhood floor area would be reduced to 90 m^2^, which is in broad agreement with the national average *usable* floor area of 92 m^2^ cited in the English Housing Survey for buildings of a similar age (Ministry of Housing, Communities & Local Government, 2018). Figure 3d provides a breakdown of the prominent façade material, see accuracy and sensitivity section for more details. Finally, Figures 3e and 3f and their insets show spatial distribution of the façade components and their total count across the neighborhood, respectively. We should clarify here that the parameters presented in Figures 3a–c have been extracted from existing building polygons maintained by the Ordnance Survey for convenience. Figures 3d shows material allocation based on the manual examination of the photos performed by two of the authors, see future work section for more information. Figures 3e,f visualizes component count estimates based on the steps described in Section 3.2.

There is, expectedly, a wider variation of component count for windows at a building level than doors. For the majority of buildings in the neighborhood, we detect a single door which is consistent with the number of doors visible on the façade of terraced and semi-detached houses to a street-level observer. (Note that in Figure 3f, the higher number of doors is often observed for corner structures where a better line-of-sight to buildings’ back doors is available.) As for windows, the detected number of street-facing windows closely follows the building type, where rows of terraced and semi-detached buildings can be seen with two to five windows, respectively, with the higher window counts observed in clusters belonging to apartments and non-residential buildings.

### Accuracy and sensitivity

Here, we briefly address some aspects of the prototype’s accuracy and sensitivity. We begin by outlining the work undertaken to quantify the accuracy of the component count.

As previously mentioned, the component counts rely on automated processing of 42,451 images. To provide a comparison, we have implemented a manual count of components within a subset of 1366 images. This subset comprises the closest image to each structure’s footprint among the 100 originally queried for each polygon. For each image, two of the authors undertook a manual count of the total number of components visible, the individual building component count, and the predominant material of the façade. We should note that manual counters have assessed their images independently and hence there has not been an expectation that their count of components or assessment of the predominant construction material visually apparent should agree. We use the average of these manual counts as an alternative in situ survey assessment against which the prototyped framework can be benchmarked. (Readers are encouraged to refer to the online Supporting Information for the comparison of manual counts by the two authors.)

**FIGURE 4:**
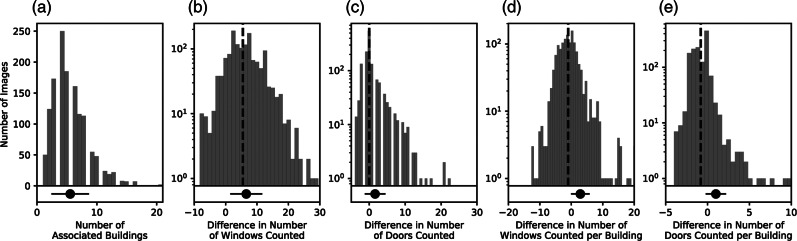
Distribution of image-level component counts by the model and its deviation from manual counts. Dashed vertical lines denote the median with the point plots indicating mean and standard deviation. Note that the axes have been truncated excluding single occurrence large values. Data from this figure are available in File S2 of the Supporting Information

Figure 4 provides detailed distribution of the modeled and manual component count variations at an image level, with Table 2 showing the summary count and sum of the components for both methods at a building level. Note that neither the figure nor the table provides a quantification of the accuracy or precision of the neural network models used. Rather, they provide a measure of the extent to which the model estimates diverge from manual human counts based on a subset of the images. (Readers should consult Dai et al. (2019) for the accuracy and precision of the neural network models used.) This is important, especially with regard to the manual building-level estimates in Table 2, which are based on single-image information.

**TABLE 2:** Summary benchmarking of the model estimated counts versus manually assigned counts

	Based on model count	Based on manual count
Number of buildings	1515	
Number of images used	42,451	1366
Mean window count per building (standard deviation)	4.65 (2.63)	4.38 (3.08)
Sum windows (standard deviation)	7248 (112)	6640
Mean door count per building (standard deviation)	1.06 (0.30)	1.09 (0.98)
Sum doors (standard deviation)	1358 (57)	1645

At an image level, the model appears to overestimate the total number of components by up to an average of one and three for per building doors and windows, respectively. This is partly due to the potential multiple counting of door and window components where constituting pixel are non-contiguous. However, the picture is slightly more nuanced, as despite the average values, the majority of the disagreements, for the exception of the total number of windows, involves an underestimation of the number of components, see median and distribution bins in Figure 4c–e. As such, the mean values are skewed by the few instances in which the model has substantially overestimated component counts. (See underlying data in File S2 of the Supporting Information provided for the values not shown in the figure.)

At a building level, the mean difference in the counts for both doors and windows suggest that, despite model simplicity, the model estimated and directly assigned counts broadly agree on an aggregated level, Table 2. The difference between the total door count estimates might appear significantly high at 20%. However, it is worth noting that such high variations, inevitable for relatively low total number of components, still only represent fractional differences in mean component count for an *average* building—compare average 1.06 doors from the model versus 1.09 doors from the manual counts. This is important, since potentially large deviations are currently implicit within a large body of material stocks studies where the use of archetypal values implies large uncertainties in total component count, especially when considering increasingly larger geographic boundaries (Lanau et al., 2019).

### Future work

In this section, both limitations of this case study and planned future work to further implement the scalable framework are outlined. We have mentioned the major challenges in truly implementing a scalable accounting framework in Section 2. The case study here using street-level imaging showcases a number of these difficulties including underestimation of overall building components due to an incomplete view of buildings.

In practical terms, one of the options for optimal circular economy is building refurbishments. This would reduce the demand for new buildings, reducing embodied impacts. However, there is a risk that if energy efficiency is not a priority, building life extension could maintain operational inefficiencies making carbon emissions from use more challenging to reduce. In building refurbishments terms, the case study presented would only be able to give a crude estimate of likely insulation and does not consider the nuances of different wall types, further work is therefore required to investigate the use of the mobile-sensing platform for the recognition of wall construction from their visual, thermal, and hyperspectral signatures. This would facilitate estimation of the surface areas that require either external or internal insulation, compared to cavity wall properties which may have already been insulated. Future work also needs to make more targeted use of thermal and hyperspectral imaging to understand heat loss patterns through the building fabric for a quantification of component material type and quality. (See Phan (2012) for an example of a prior attempt at incorporating thermal imaging.) Finally, parallel work is under way at the time of writing this article, focusing on classifying buildings into archetypes for the United Kingdom, something that is currently lacking in a specialized sense for the UK building stock. This will be valuable, and we aim to integrate these archetypes within the framework presented here as set out in Section 2.4.

## CONCLUSIONS

In this paper we have demonstrated the application of a framework based on mobile sensing and machine learning to automate the estimation of building stock components and materials. This represents an advance on existing bottom-up accounting of building stocks that rely entirely on archetypes. Indeed, the semantic reconstruction of the built environment in 3D would offer multiple benefits. First, accounting exercises based on such a framework provide building-specific registers of components. At its bare minimum, mobile sensing provides high-fidelity individualized measurement of buildings. Given access to relevant archetypes for an area, the framework can also provide a spatially explicit archetype matching method that provides the same level of information as archetypal average characteristic estimates. Fully functioning, however, the framework enables building-level detection of various facade components and their material, for example, windows and wall, and an understanding of both what is available to *urban mine* in the future based on building-level measurements, as well as indicating likely future material demands for building refurbishments. Additionally, the combined building-specific information on components, their dimensions, and constituting material allow for a much more accurate and spatially resolute quantification of material stock which is currently partially achieved through remote sensed information.

## Supplementary Information

**Supporting Information S1**: This Supporting Information S1 is a zip archive that includes this summary file and four additional files: File S1, provided as a docx file, outlines a brief demonstration of the use of the models by Dai et al. (2019) using Google Street View images from different cities and a comparison of the manual components counts performed by two of the authors across 1366 images. This details the extent of disagreement between the manually counted total number of windows and doors and per building count of these components. File S2, provided as a xlsx file, contains human readable numerical approximation of the values embedded within the figures throughout the manuscript and the supporting information. File S3, provided as csv file, contains the underlying data used in the case study analysis and creating the figures in the form of a image-by-building schedule of structures and modelled component counts accompanied (100x1515 rows). File S4, provided as csv file, contains the underlying data used in the case study analysis and creating the figures in the form of an image-wise schedule of manual component counts (1366 rows). (ZIP 6.01 MB)
