# Supplementary material for: A scalable data collection, characterization, and accounting framework for urban material stocks
Source: J Ind Ecol. 2021 Sep 25;26(1):58–71. doi: 10.1111/jiec.13198 (PMC13090203; doi:10.1111/jiec.13198)
Supplement: Supplementary file 1 — Supporting Information S1: This Supporting Information S1 is a zip archive that includes this summary file and four additional files: File S1, provided as a docx file, outlines a brief demonstration of the use of the models by Dai et al. (2019) using Google Street View images from different cities and a comparison of the manual components counts performed by two of the authors across 1366 images. This details the extent of disagreement between the manually counted total number of windows and doors and per building count of these components. File S2, provided as a xlsx file, contains human readable numerical approximation of the values embedded within the figures throughout the manuscript and the supporting information. File S3, provided as csv file, contains the underlying data used in the case study analysis and creating the figures in the form of a image-by-building schedule of structures and modelled component counts accompanied (100x1515 rows). File S4, provided as csv file, contains the underlying data used in the case study analysis and creating the figures in the form of an image-wise schedule of manual component counts (1366 rows). (ZIP 6.01 MB) [file 44498_2022_2601005_MOESM1_ESM.zip › S1_arbabi_et_al._(2021)_jie_si_v2.1.docx]

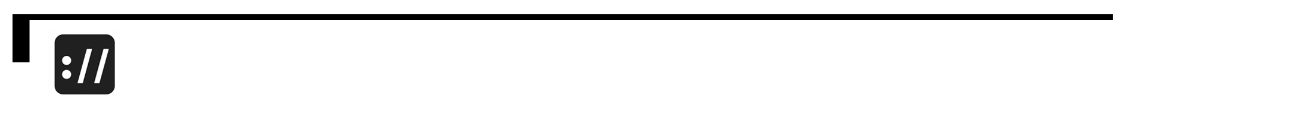


SUPPORTING INFORMATION FOR:

Hadi Arbabi, Maud Lanau, Xinyi Li, Gregory Meyers, Menglin Dai, Martin Mayfield & Danielle Densley Tingley (2021.) A Scalable Data Collection, Characterization, and Accounting Framework for Urban Material Stocks. *Journal of Industrial Ecology.*


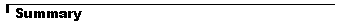


S1, provided as a docx file, outlines a brief demonstration of the use of the models by Dai et al. (2019) using Google Street View images from different cities and a comparison of the manual components counts performed by two of the authors across 1366 images. This details the extent of disagreement between the manually counted total number of windows and doors and per building count of these components.


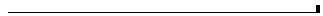


The data used to create the figures in the main manuscript and this supporting information have been provided in two separate files as comma separated values. Approximations of the values directly from the figures are also provided in a separate spreadsheet.

**Google Street View trial**

We have noted within the manuscript that there exists a universality of features to envelope components such as doors and windows that should allow for the deployment of any one model trained on segmenting street-level mobile sensed imagery to function across contexts with minimal modification and retraining. Had Dai et al.’s model ensemble (2019) been trained to detect architectural archetypes and vintage, ie, age cohort, maintaining model generalizability across various architectural contexts would have required near constant improvements to the training of its individual models in terms of geographic and national inclusion. The geometry and characteristics of components such as doors and windows are, however, more universal than they might initially appear (Zhu et al., 2020). Here we have included a demonstration of Dai et al.’s model segmenting images extracted from Google Street View from a number of different countries to demonstrate this point, Figure SI1.

| 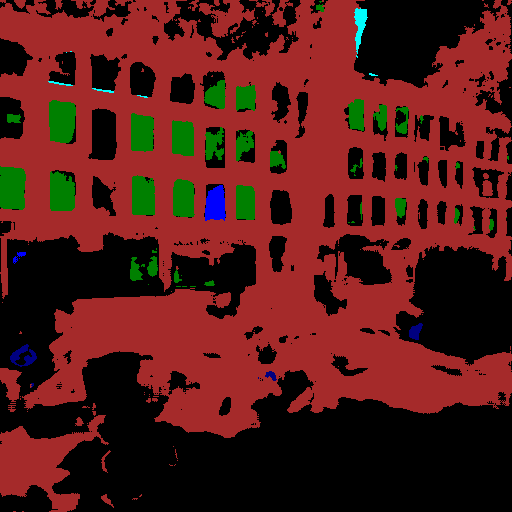 | 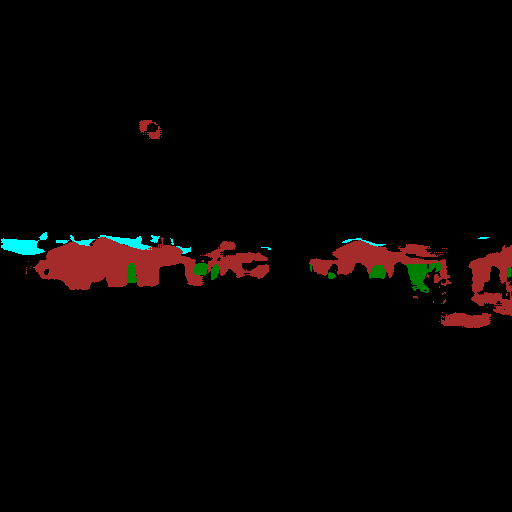 | 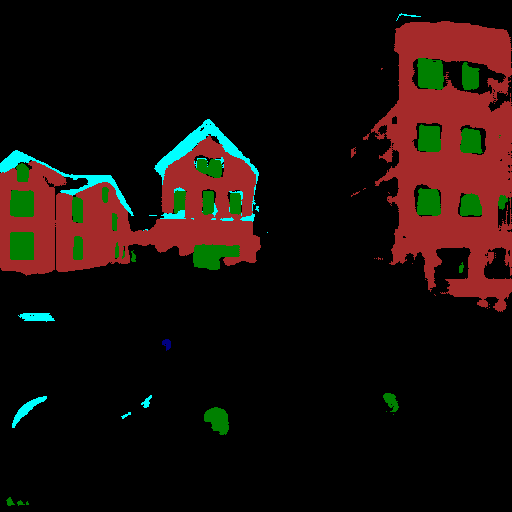 | 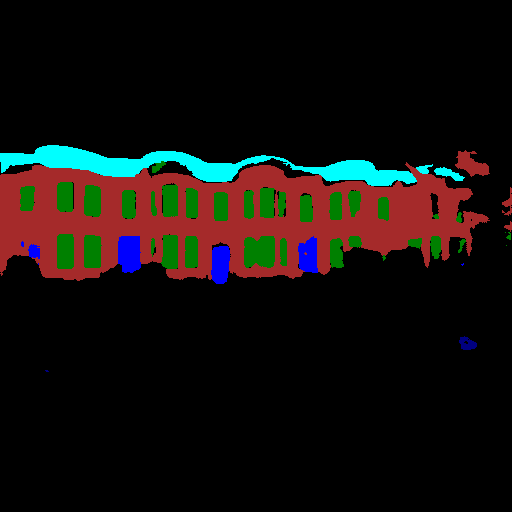 |
| --- | --- | --- | --- |
| 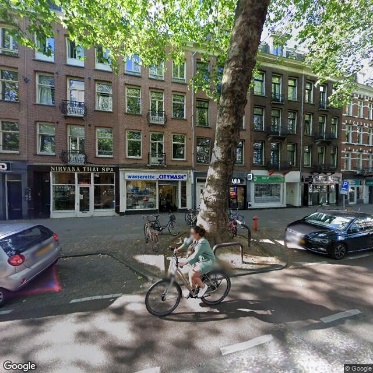 | 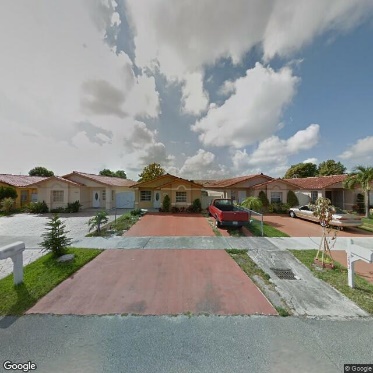 | 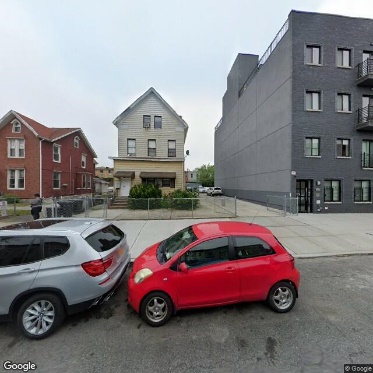 | 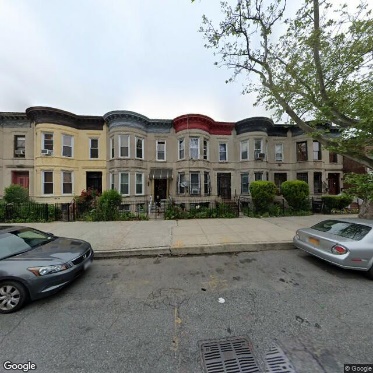 |
| Amsterdam, The Netherlands | Hialeah, Florida | New York City, New York | New York City, New York |
| 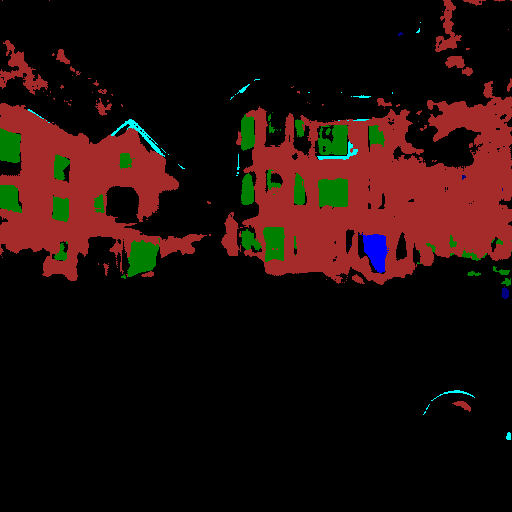 | 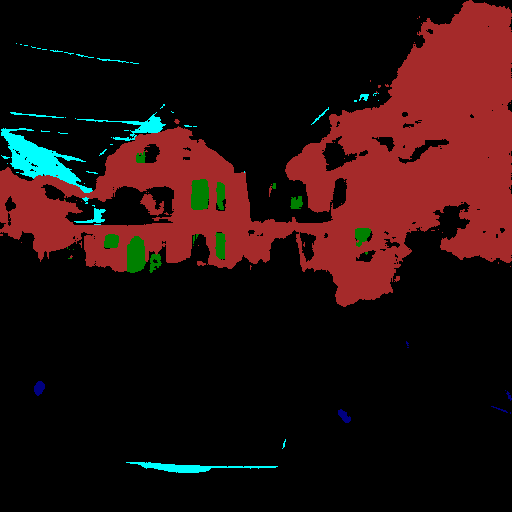 | 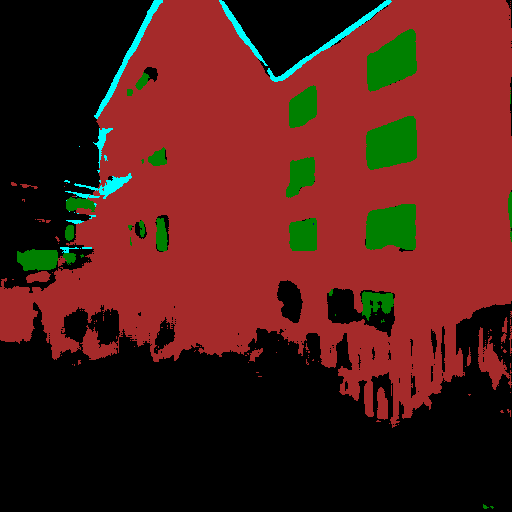 | 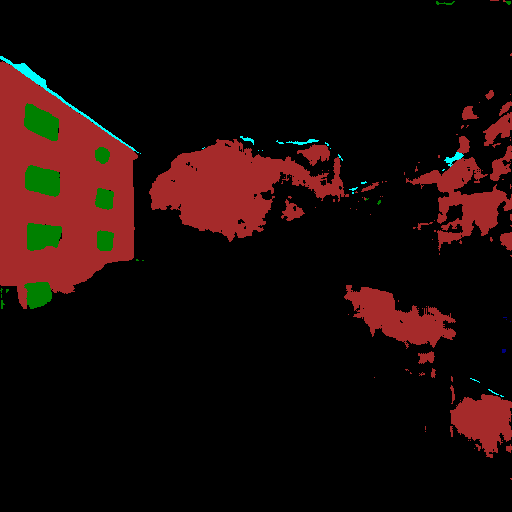 |
| 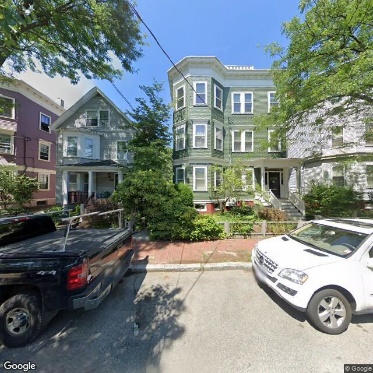 | 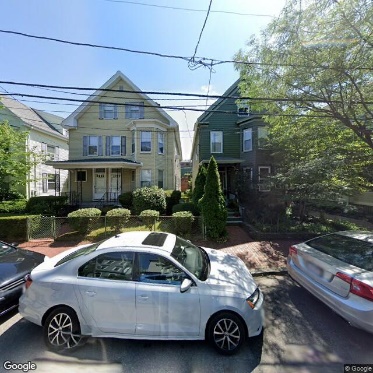 | 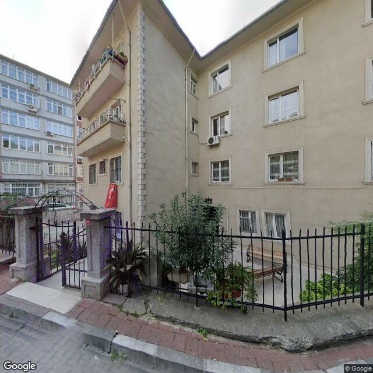 | 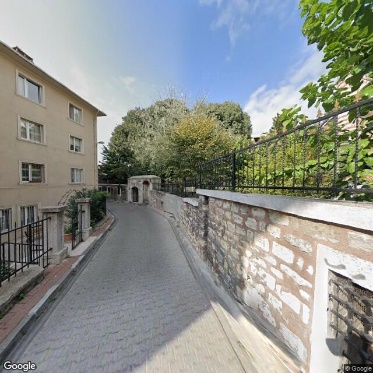 |
| Cambridge, Massachusetts | Cambridge, Massachusetts | Istanbul, Turkey | Istanbul, Turkey |

Figure SI1. Prediction outputs for envelope components using Google Street View images from 5 different cities in 3 different countries and Dai et al. model as originally trained on multi-spectral advanced research vehicle imaging in Sheffield, UK.

For perfect performance, the model would ideally have to be fine-tuned to the context of interest to address issues from the variation in the source of the input images to the weather conditions captured across the training set. Although true, it is clear form panels in Figure SI1 that the model’s output without such tweaking, while far from ideal, is still functional demonstrating the generalizability of the façade components across national and geographic contexts based on a small training set of residential buildings in Sheffield, UK.

**Manual component count**

Here, we briefly outline a comparison between the two separate manual counts of components undertaken by two of the authors. As mentioned in the main manuscript, the manual accounting of the components is performed for a 1366-image subset of the images processed by the models. The subset is the selection of the single closes image to each building. Since there are 1515 polygons but only 1366 images, there is some overlap in coverage. For each image, the manual accounting includes counting the total number of windows and doors visible, the count of buildings closest to the vehicle, the count of doors and windows that are visible for these and/or are certain to exist, and the predominant material of the façade. Each image is counted separately by two of the authors. The variation in the counts performed by the authors is summarized in Figure SI2. In summary, the manual count of windows and doors differ an average of 2.4 and 0.9 components, respectively. For per building counts, this disagreement average 0.5 windows and 0.2 doors.


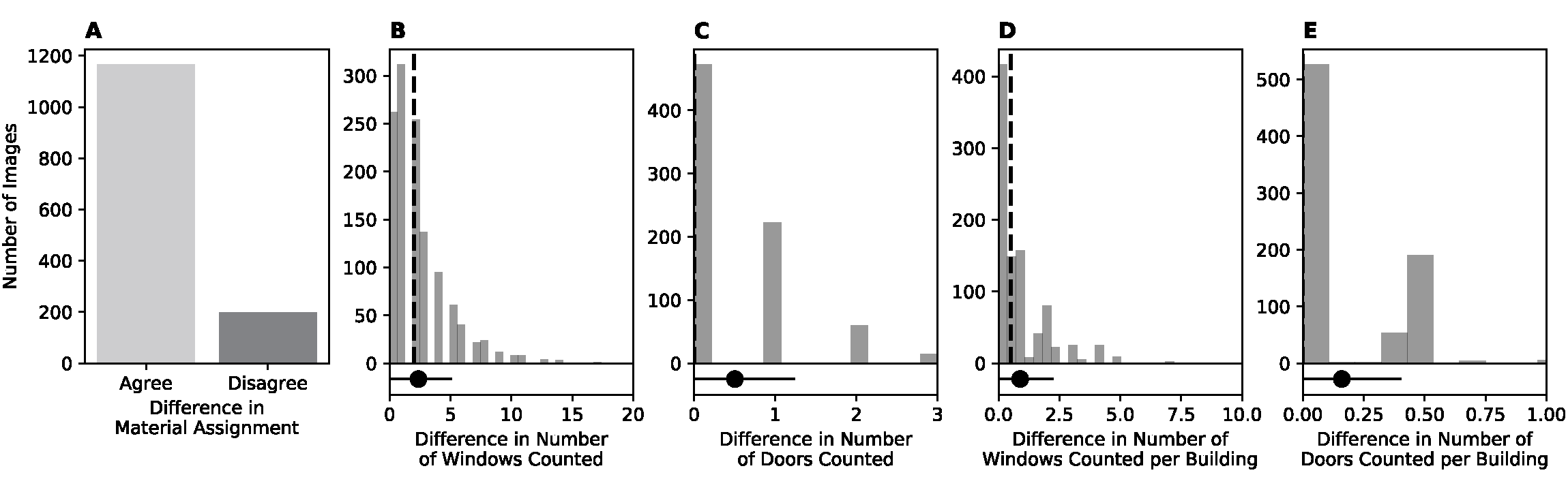


Figure SI2. Plots showing the variation in counts between the two authors where dashed vertical lines denote the median with the point plots indicating mean and standard deviation. Note that axes have been truncated exclusion single instances of very large values that distort the scale. In most images there is agreement between the examiners as to the prominent building material (A). This includes instances where the both examiners are unsure as to the nature of the constituting material. In roughly 15% of images material detected varies between the two examiners. These almost exclusively involve disagreements in differentiating brick and stone structures. The variation in total window count are more pronounced (B). In a 566 majority of the images, the authors disagree by 1 or 2 windows followed by 262 images where there is no disagreement in the counts. Note that these agreements do not include images where both examiners agree that there are no windows visible. For total door count, there is agreement in the counts for 473 images (C). In per building window counts, there is consensus in counts for only 389 images with a further 180 having differences of less than 1 window (D). Finally, there is agreement in per building door counts for 527 images (E).
