# Supplementary material for: A scalable data collection, characterization, and accounting framework for urban material stocks
Source: J Ind Ecol. 2021 Sep 25;26(1):58–71. doi: 10.1111/jiec.13198 (PMC13090203; doi:10.1111/jiec.13198)

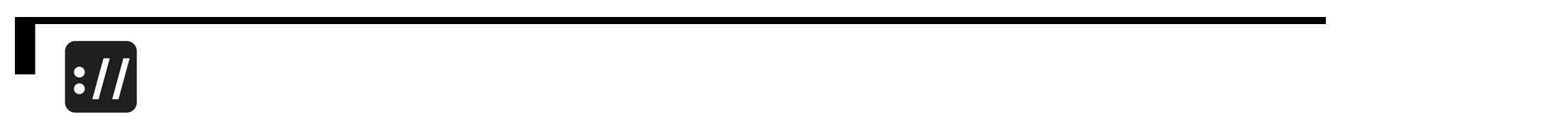


SUPPORTING INFORMATION FOR:

Hadi Arbabi, Maud Lanau, Xinyi Li, Gregory Meyers, Menglin Dai, Martin Mayfield & Danielle Densley Tingley (2021.) A Scalable Data Collection, Characterization, and Accounting Framework for Urban Material Stocks. *Journal of Industrial Ecology.*


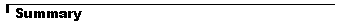


This Supporting Information S1 is a zip archive that includes this summary file and four additional files: File S1, provided as a docx file, outlines a brief demonstration of the use of the models by Dai et al. (2019) using Google Street View images from different cities and a comparison of the manual components counts performed by two of the authors across 1366 images. This details the extent of disagreement between the manually counted total number of windows and doors and per building count of these components. File S2, provided as a xlsx file, contains human readable numerical approximation of the values embedded within the figures throughout the manuscript and the supporting information. File S3, provided as csv file, contains the underlying data used in the case study analysis and creating the figures in the form of a image-by-building schedule of structures and modelled component counts accompanied (100x1515 rows). File S4, provided as csv file, contains the underlying data used in the case study analysis and creating the figures in the form of an image-wise schedule of manual component counts (1366 rows).


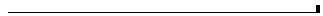

Supplement: Supplementary file 1 — Supporting Information S1: This Supporting Information S1 is a zip archive that includes this summary file and four additional files: File S1, provided as a docx file, outlines a brief demonstration of the use of the models by Dai et al. (2019) using Google Street View images from different cities and a comparison of the manual components counts performed by two of the authors across 1366 images. This details the extent of disagreement between the manually counted total number of windows and doors and per building count of these components. File S2, provided as a xlsx file, contains human readable numerical approximation of the values embedded within the figures throughout the manuscript and the supporting information. File S3, provided as csv file, contains the underlying data used in the case study analysis and creating the figures in the form of a image-by-building schedule of structures and modelled component counts accompanied (100x1515 rows). File S4, provided as csv file, contains the underlying data used in the case study analysis and creating the figures in the form of an image-wise schedule of manual component counts (1366 rows). (ZIP 6.01 MB) [file 44498_2022_2601005_MOESM1_ESM.zip › A Summary of the Supporting Information.docx]
